# Ossification of the Nuchal Ligament With Text Neck Syndrome

**DOI:** 10.7759/cureus.115774

**Published:** 2026-09-04

**Authors:** Cheuk Wing Chiang, Eric Chun-Pu Chu

**Affiliations:** 1 Chiropractic and Physiotherapy Centre, New York Medical Group, Hong Kong, CHN

**Keywords:** cervical instability, cervical spondylosis, chiropractic, forward head posture, ossification of the nuchal ligament, ossification of the posterior longitudinal ligament, text neck syndrome

## Abstract

Ossification of the nuchal ligament (ONL) is a common but frequently overlooked radiographic finding on lateral cervical spine imaging, characterized by ectopic bone formation within the nuchal ligament, most often at the C4/5 and C5/6 levels. Although usually asymptomatic, ONL has been associated with cervical spondylosis, segmental instability, and, importantly, a markedly increased risk of coexisting ossification of the posterior longitudinal ligament (OPLL), a condition with potential for progressive myelopathy. We conducted a narrative review of the PubMed-indexed literature on the anatomy, epidemiology, classification, and clinical significance of ONL, illustrated with the case of a 61-year-old female who presented with neck stiffness and headache in the context of habitual mobile phone use of 10 hours per day. Lateral cervical radiography demonstrated loss of normal cervical lordosis, moderate degenerative disc disease most severe at C5/6, and ossification of the ligamentum nuchae posterior to C4 and C5, consistent with moderate cervical spondylosis. She improved with four weeks of care. This case illustrates the classic radiographic and clinical profile of ONL in the setting of reported prolonged device use and forward head posture, highlighting a possible contributing mechanical factor. Given the strong statistical link between ONL and OPLL, primary healthcare providers who incidentally identify ONL on lateral cervical radiographs should consider it a clinical marker warranting careful neurological evaluation. Advanced cross-sectional imaging (CT or MRI) is not automatically indicated for isolated, asymptomatic ONL, but should be considered when neurological signs or symptoms, suspected myelopathy, progressive deficits, trauma, or specific manual therapy safety considerations arise.

## Introduction and background

The nuchal ligament (ligamentum nuchae) is a strong fibroelastic septum extending from the external occipital protuberance to the spinous process of C7, providing dynamic support to the head and cervical spine during flexion-extension [1]. Its lower segments, particularly the attachments at C6 and C7, sustain peak mechanical strain during neck flexion [1]. Chronic repetitive tensile microtrauma at these fibro-osseous junctions has been proposed as a key mechanism that may trigger ectopic ossification, a process known as ossification of the nuchal ligament (ONL) [1]. On lateral cervical radiographs and computed tomography (CT), ONL appears as a smooth-contoured, ovoid, rod-shaped, or segmented radiodense structure posterior to the spinous processes, most frequently at C4/5 and C5/6 [2,3]. To synthesize current evidence on this condition, this PubMed-based narrative review aims to summarize the anatomical basis, epidemiology, classification, and clinical significance of ONL-particularly its association with coexisting ossification of the posterior longitudinal ligament (OPLL) and manual therapy safety-complemented by an illustrative case encounter.

ONL is a common, usually incidental finding: it has been reported in up to 49.7% of patients with cervical spondylosis undergoing plain radiography [2] and in 9.14% of patients undergoing cervical CT for unrelated indications [3]. Established risk factors include advancing age, male sex, obesity, and impaired glucose metabolism, which similarly predispose to ossification of the anterior and posterior longitudinal ligaments [4]. More recently, cephalometric evidence has demonstrated an association between ONL and increased forward head posture [5], suggesting that sustained postural strain from activities, such as prolonged mobile device use, represents a possible, though unproven, biomechanical contributor to its development [5].

Although ONL itself is rarely symptomatic, it correlates with pathological changes of the cervical spine, including intervertebral disc degeneration and osteophyte formation at the level of ossification, and with segmental instability [6]. Of particular clinical importance is its association with OPLL: the prevalence of OPLL is significantly higher in patients with ONL than in those without it [7], and a meta-analysis of 8429 participants found that ONL nearly quadruples the odds of coexisting OPLL (odds ratio 3.84) [8]. Despite growing epidemiologic evidence linking ONL to cervical spondylosis, segmental instability, and coexisting OPLL [6-8], a key gap remains in the primary musculoskeletal literature: ONL is frequently dismissed as a benign, incidental curiosity, and there is a lack of synthesized guidance for first-contact clinicians regarding its diagnostic interpretation, clinical triage, and manual therapy safety implications. Because coexisting OPLL carries a risk of progressive cervical myelopathy and alters the safety profile for cervical spinal interventions, primary care providers require a clear framework to identify when incidental ONL warrants heightened clinical correlation versus advanced cross-sectional imaging. To address this literature gap, this article provides a narrative review of the anatomy, epidemiology, classification, and clinical significance of ONL, integrated with a representative case illustration to offer practical, evidence-informed guidance for primary musculoskeletal practice.

## Review

A 61-year-old female presented to a chiropractic clinic with a six-month history of worsening neck stiffness, dull occipital headache, and intermittent aching across the upper trapezius musculature. She reported no history of acute trauma. A detailed history revealed several daily lifestyle and occupational stressors: she worked full-time in a restaurant requiring frequent forward-bending tasks and prolonged standing, slept habitually on two firm pillows that held her head in sustained flexion, and reported non-work mobile phone use averaging 10 hours per day in a head-down posture.

On physical examination, she exhibited pronounced forward head posture and restricted active cervical range of motion, particularly in flexion (reproducing posterior neck tightness) and right rotation. Cervical compression and Spurling’s tests were negative for radicular arm pain, and upper extremity deep tendon reflexes, motor strength, and sensation were intact bilaterally, with no myelopathic signs (negative Hoffmann's and Babinski’s signs). Lateral cervical radiography was obtained as part of her initial structural evaluation (Figure 1).

**Figure 1:**
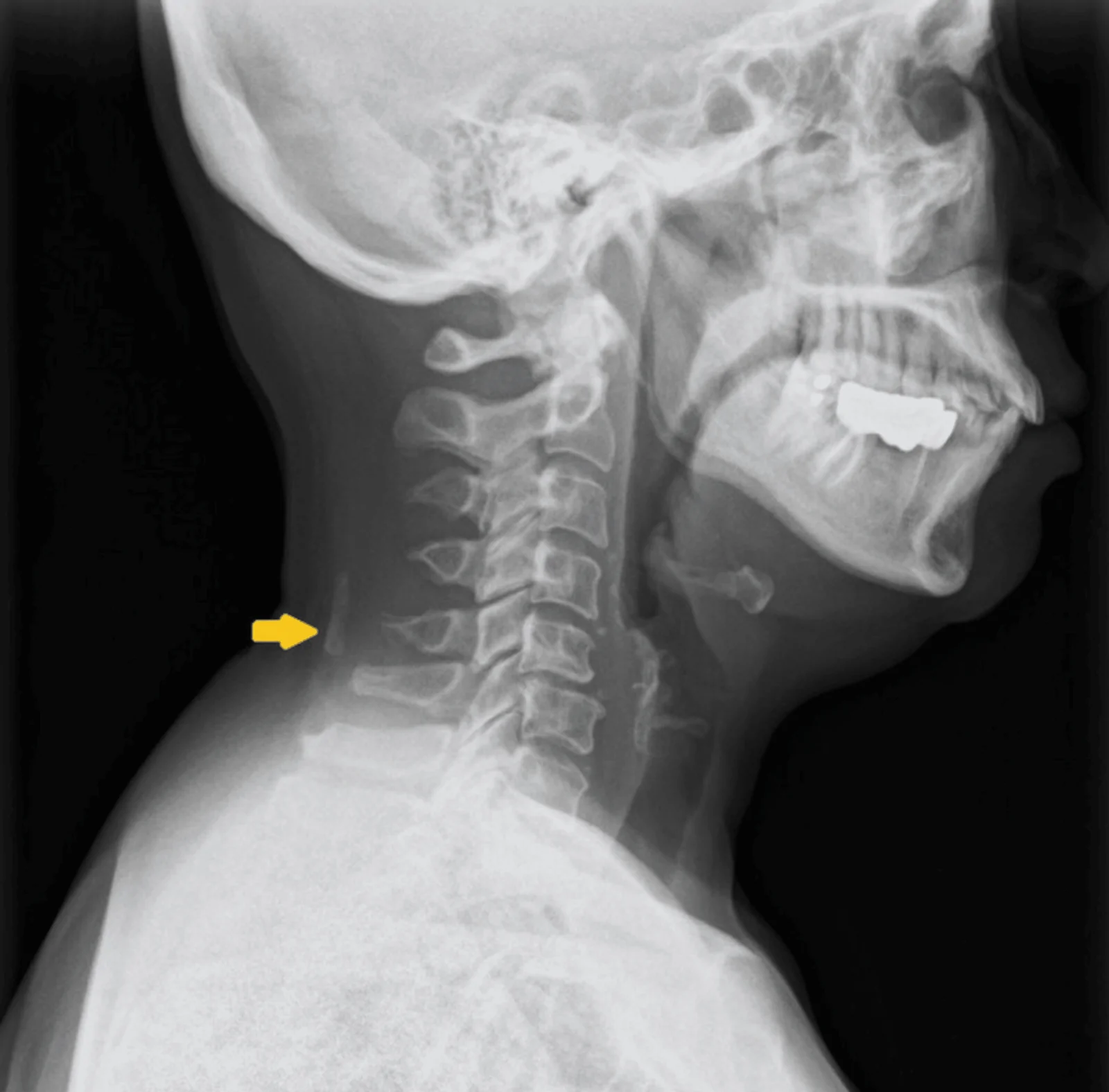
Lateral cervical radiograph demonstrating loss of normal cervical lordosis, moderate degenerative disc disease with loss of disc height and marginal osteophytosis most severe at C5/6, mild facet and uncovertebral joint degeneration, and ossification of the ligamentum nuchae posterior to C4 and C5 (yellow arrow).

Radiographic findings included straightening of the normal cervical lordosis, which may reflect antalgic posture, pain-related muscular guarding, or positioning during film acquisition, without scoliosis. Moderate degenerative disc disease was present, with loss of disc height and marginal osteophytosis most severe at C5/6, and the facet and uncovertebral joints were mildly degenerated. Ossification of the ligamentum nuchae was identified posterior to C4 and C5, corresponding to the local type of ONL as classified in prior radiographic series [2]. The C1/2 articulation was mildly degenerated, the craniovertebral junction was normal, and no prevertebral soft tissue abnormality, reduced bone density, or cervical rib was observed. The overall impression was moderate cervical spondylosis.

The patient was managed with a structured, multimodal conservative care plan delivered three times weekly over four weeks. Given her age, degenerative changes, and the incidental presence of ONL, high-velocity, low-amplitude rotational thrust manipulation was provided to improve segmental mobility, coupled with post-isometric relaxation stretching directed at hypertonic upper trapezius and levator scapulae muscles. Soft tissue release (instrument-assisted soft tissue mobilization (IASTM)) and shockwave therapy were applied to symptomatic posterior cervical and occipital trigger points.

Care was integrated with ergonomic education and postural retraining: the patient was advised to modify her sleeping setup to a single supportive cervical contour pillow and instructed on eye-level mobile device positioning with scheduled postural rest breaks during work and personal screen time. Following four weeks of treatment, her neck stiffness and headache resolved, and her pain score decreased from 7/10 to 1/10 on the Numeric Rating Scale (NRS).

Review of the literature

Methods and Literature Search Strategy 

As a case report and narrative review, a structured literature search was conducted to synthesize current evidence on ONL and contextualize the presented case. Primary electronic databases searched included PubMed, Scopus, and the Index to Chiropractic Literature (ICL) for peer-reviewed studies published between January 2000 and December 2025.

The search strategy utilized Boolean operators combining the following terms: ("ossification of the nuchal ligament" OR "nuchal ligament ossification" OR "ONL") AND ("ossification of the posterior longitudinal ligament" OR "OPLL" OR "cervical spondylosis" OR "chiropractic" OR "spinal manipulation" OR "conservative management"). Eligibility was limited to English-language original research, observational studies, systematic/narrative reviews, meta-analyses, and relevant clinical case series examining ONL epidemiology, classification, biomechanics, risk factors, or non-surgical treatment outcomes.

Conservative Management Outcomes in Symptomatic ONL 

Literature evaluating conservative clinical outcomes specifically dedicated to isolated ONL is relatively sparse, as ONL is routinely managed within the broader spectrum of cervical spondylosis, myofascial pain, and cervical radiculopathy. However, existing evidence indicates that symptomatic patients lacking myelopathic compromise respond favorably to multimodal conservative interventions. Non-surgical approaches reported in the literature include low-force segmental mobilizations, post-isometric relaxation stretching, soft-tissue myofascial release, and passive physical modalities. Observational data demonstrate that these strategies effectively reduce pain intensity and functional disability (measured via NRS and Neck Disability Index) by relaxing paraspinal muscle guarding and restoring regional mobility, even though the heterotopic ossification itself remains anatomically unchanged. In conservative spine care, high-velocity rotational thrust manipulation is frequently substituted with non-thrust mobilization techniques - particularly when ONL co-occurs with extensive spondylosis or suspected OPLL - to minimize mechanical stress on adjacent hypermobile segments. Furthermore, active patient education targeting ergonomic modifications and postural retraining plays an essential long-term role in managing symptom recurrence.

Anatomy and Biomechanics of the Nuchal Ligament

The ligamentum nuchae is a triangular fibroelastic membrane in the posterior midline of the neck, extending from the external occipital protuberance and nuchal crest to the spinous process of C7, where it becomes continuous with the supraspinous ligament of the thoracic and lumbar spine [1]. Histologically, its lower cervical portion contains a higher proportion of dense collagenous tissue and fewer elastic fibers than its upper portion, an architecture that favors load-bearing over pure elastic recoil and renders the C6-C7 attachment particularly susceptible to chronic tensile microtrauma during repetitive flexion-extension movements [1]. Sustained or repetitive strain at these fibro-osseous junctions is thought to incite an ossific response analogous to enthesopathy elsewhere in the musculoskeletal system, which over time produces the radiodense structures seen radiographically as ONL, predominantly at the C4/5 and C5/6 levels where cervical mobility, and therefore ligamentous strain, is greatest [2,3].

Epidemiology, Classification, and Risk Factors

Wang et al. proposed a five-type radiographic classification of ONL: local (focal), continuous, segmental, mixed, and unclassified, based on morphology on lateral cervical radiographs, with the local type being most common [2]. In their series of patients with cervical spondylosis, ONL was identified in 49.7% of cases, most frequently at C4/5 and C5/6 [2]. Population-based CT studies report a lower but still substantial incidence of approximately 9.14% among patients undergoing cervical CT for unrelated indications, likely reflecting differences between screened spondylosis populations and general imaging cohorts [3]. Established risk factors include older age, male sex, obesity, and impaired glucose metabolism [4], while a growing body of evidence implicates chronic forward head posture, as commonly produced by prolonged smartphone or computer use, as a contributing biomechanical stressor: a cephalometric study found significantly greater forward head posture, by C0-C1 distance and craniocervical angle, in patients with ONL than in those without [5]. This observation is consistent with the broader concept of text neck syndrome, in which sustained cervical flexion during device use imposes cumulative structural strain on posterior cervical soft tissues [9].

Association With Cervical Spondylosis, Instability, and OPLL

Beyond simple prevalence, the location of ONL correlates meaningfully with other degenerative changes in the cervical spine. Wang et al. found that osteophyte height and intervertebral disc degeneration grading were significantly greater at the level of ONL than at adjacent segments, and that ONL commonly corresponded to the most stenotic level of the spinal canal in patients with cervical spondylosis [6]. Radiographic and dynamic imaging studies have further shown that patients with ONL demonstrate greater sagittal malalignment and segmental angular and horizontal displacement on flexion-extension views than matched controls, indicating an association with cervical instability [10]. Similarly, larger ONL size correlates with reduced active range of motion, more severe cervical radiculopathy, and greater neural foraminal stenosis, linking the radiographic finding to tangible clinical symptoms such as neck stiffness [11].

The most clinically significant association of ONL, however, is with OPLL. Kim et al. reported an OPLL prevalence of 64.7% in patients with ONL compared with 16.1% in those without it, with larger ONL morphologies (rod and segmented types) associated with more extensive, multilevel OPLL [7]. A separate CT-based study similarly found OPLL nearly 2.5 times more likely in patients with ONL [12]. These findings were corroborated by a meta-analysis of 10 cohort studies encompassing 8429 participants, which demonstrated an odds ratio of 3.84 for coexisting OPLL in the presence of ONL, independent of age, sex, and body mass index [8]. Because OPLL is a progressive condition capable of producing cervical myelopathy and carries specific relevance to the safety of cervical spinal manipulation, and because ONL resection during surgical decompression has itself been linked to reduced long-term cervical stability [13], the identification of ONL on a routine lateral radiograph, as in the case presented here, should prompt consideration of further evaluation, including CT, when clinical features raise concern for coexisting OPLL or instability.

Selected studies characterizing the association between ONL and OPLL are summarized in Table 1.

**Table 1:** Selected studies characterizing the association between ossification of the nuchal ligament (ONL) and ossification of the posterior longitudinal ligament (OPLL).

Study	Design/population	Key findings
Kim et al. (2015) [7]	Retrospective lateral radiograph review	OPLL prevalence 64.7% with ONL vs. 16.1% without; larger (rod/segmented) ONL types linked to more extensive, multilevel OPLL.
Kim et al. (2019) [12]	Retrospective CT review, 297 patients	OPLL nearly 2.5 times more frequent in patients with ONL; no correlation between ONL and OPLL length.
Zhang et al. (2021) [8]	Meta-analysis, 10 cohort studies, 8429 participants	Odds ratio 3.84 for coexisting OPLL in the presence of ONL, independent of age, sex, and body mass index.
Chen et al. (2025) [13]	Long-term follow-up after modified laminoplasty	Resection of ONL, especially involving two or more cervical segments, associated with increased long-term cervical instability.

Discussion

The case presented here exemplifies the typical radiographic and demographic profile of ONL described in the literature: an older adult with cervical spondylosis, ossification localized to the C4/5 region, the segments of greatest cervical mobility, and coexisting disc degeneration and osteophytosis most severe at the immediately adjacent C5/6 level [2,6]. The patient's reported habitual mobile phone use of 10 hours per day is a plausible contributing factor, given recent cephalometric evidence linking ONL to pronounced forward head posture [5], and parallels our group's previous descriptions of postural cervical syndromes. Because objective postural parameters (such as the craniocervical angle or C0-C1 distance) were not formally measured in this patient, and because the exact onset and temporal development of her ONL remain unknown, no definitive biomechanical pathway or causal link can be inferred from this case. While self-reported daily device use and forward-leaning occupational tasks represent potential sources of cumulative mechanical stress, a single case report cannot validate a postural-mechanical mechanism. This observation should instead be viewed strictly as hypothesis-generating, warranting evaluation in prospective studies that utilize objective postural metrics and longitudinal imaging.

For first-contact musculoskeletal providers, the principal clinical value of recognizing ONL lies not in the finding itself, which is usually benign, but in its statistical association with OPLL and cervical instability [7,8,10]. In the patient described here, follow-up or advanced imaging was not performed after treatment because her presentation lacked myelopathic features, and she achieved rapid, complete symptom resolution. From a clinical management standpoint, repeating plain radiographs or ordering cross-sectional imaging in an asymptomatic patient is generally not recommended to avoid unnecessary ionizing radiation exposure and medical expenditure. However, from a scientific perspective, obtaining post-treatment and long-term delayed imaging could have provided more definitive, authentic evidence regarding the structural stability, morphological changes, or progression of the ONL following conservative care.

It is important to emphasize that an incidental finding of ONL on plain radiographs is a radiographic marker rather than an established contraindication to cervical manipulation. Isolated, asymptomatic ONL does not preclude manual therapy. However, clinicians must distinguish incidental ONL from high-risk clinical scenarios: when ONL co-occurs with clinical features suggestive of cervical myelopathy (e.g., gait disturbance, hand clumsiness, hyperreflexia), progressive radiculopathy, or suspected segmental instability, the statistical association with coexisting OPLL [8] warrants caution. In such symptomatic or high-risk contexts, advanced cross-sectional imaging (CT or MRI) should be pursued to evaluate canal dimensions and rule out cord compromise prior to initiating high-velocity thrust procedures. This is consistent with the precedent set by an isolated case report of incidentally discovered ONL prompting further clinical evaluation [14], and reinforces the broader principle that incidental radiographic findings in musculoskeletal practice can carry systemic or structural implications well beyond their immediate appearance.

This narrative review has several limitations inherent to its design, including reliance on published observational studies predominantly from Asian populations, in whom ONL and OPLL are more extensively studied, which may limit generalizability to other ethnic groups. Similarly, our illustrative case is a single, retrospectively described observation without short-term or delayed post-treatment imaging to objectively verify ONL structural stability, monitor potential OPLL development, or confirm anatomical changes following symptom resolution. Future research should prioritize prospective, cohorts that correlate ONL morphology on plain radiographs with device-use patterns, postural parameters, and outcomes of conservative care, and should evaluate whether targeted CT screening for OPLL in patients with extensive or multilevel ONL, particularly those with additional risk factors such as older age, male sex, or obesity [4,8], meaningfully changes clinical management or manipulation safety decisions in primary musculoskeletal practice. The epidemiological and clinical data synthesized in this review rely heavily on cohorts from East Asian populations, where the prevalence of heterotopic ligamentous ossifications (both ONL and OPLL) is documented to be substantially higher (up to 49.7%) than in Western or Caucasian populations [2,3]. Caution must therefore be exercised when generalizing these prevalence rates and risk correlations to non-Asian clinical demographics. The quantitative effect estimates cited in this narrative review (e.g., odds ratios and prevalence percentages with 95% CIs) are derived from external observational and cross-sectional studies. Because observational designs are inherently susceptible to selection bias, recall bias, and unmeasured confounding (e.g., age-related degenerative co-variables), these statistical associations should not be interpreted as definitive causal proof. Clinicians should view reported effect sizes as probabilistic indicators to guide clinical triage rather than absolute diagnostic guarantees.

## Conclusions

ONL is a common, usually asymptomatic radiographic finding most often located at the C4/5 and C5/6 levels, but it carries meaningful clinical significance through its association with cervical spondylosis, segmental instability, and a markedly increased risk of coexisting OPLL. The case presented here illustrates the classic profile of ONL co-occurring with prolonged mobile phone use and postural strain, highlighting device-related forward head posture as a possible contributing factor worth further prospective study. Primary healthcare providers who identify ONL on lateral cervical radiographs should regard it as more than an incidental curiosity, and should consider closer clinical correlation. Future prospective studies evaluating conservative interventions for ONL should incorporate standardized baseline and delayed post-treatment imaging protocols to establish authentic objective evidence regarding structural changes, stability, and long-term clinical outcomes.
